# *Communication and access to healthcare*: Experiences of Aboriginal and Torres Strait Islander people managing pain in Queensland, Australia

**DOI:** 10.3389/fpain.2022.1041968

**Published:** 2022-12-06

**Authors:** Christina Maresch Bernardes, Kushla Houkamau, Ivan Lin, Marayah Taylor, Stephen Birch, Andrew Claus, Matthew Bryant, Renata Meuter, Jermaine Isua, Paul Gray, Joseph P Kluver, Corey Jones, Stuart Ekberg, Gregory Pratt

**Affiliations:** ^1^ Aboriginal and Torres Strait Islander Health Research Program, QIMR Berghofer Medical Research Institute, Brisbane, QLD, Australia; ^2^ Western Australian Center for Rural Health, University of Western Australia, Geraldton, WA, Australia; ^3^ North Queensland Persistent Pain Management Service, Townsville Hospital and Health Service, Townsville, QLD, Australia; ^4^ Centre for Business and Economics of Health, Faculty of Business, St Lucia, QLD, Australia; ^5^ Tess Cramond Pain and Research Centre, Metro North Hospital and Health Service, Brisbane, QLD, Australia; ^6^ School of Psychology and Counselling, Faculty of Health, Brisbane, QLD, Australia; ^7^ Aboriginal and Torres Strait Islander Health Division, Cultural Capability Services, Brisbane, QLD, Australia; ^8^ Persistent Pain Clinic, Metro South Hospital and Health Service, Brisbane, QLD, Australia

**Keywords:** communication, Aboriginal, Torres Strait Islander, pain, access to healthcare

## Abstract

**Background:**

Pain management requires a multidisciplinary approach and a collaborative relationship between patient-provider in which communication is crucial. This study examines the communication experiences of Aboriginal and Torres Strait Islander patients and Aboriginal and Torres Strait Islander Hospital Liaison Officers (ATSIHLOs), to improve understanding of how pain is managed in and through patient-health professional communication.

**Methods:**

This qualitative study involved a purposive sample of patients attending three persistent pain clinics and ATSIHLOs working in two hospitals in Queensland, Australia. Focus groups and in-depth interviews explored the communication experiences of patients managing pain and ATSIHLOs supporting patients with pain. This study adopted a descriptive phenomenological methodology, as described by Colaizzi (1978). Relevant statements (patient and ATSIHLOs quotes) about the phenomenon were extracted from the transcripts to formulate meanings. The formulated meanings were subsequently sorted into thematic clusters and then integrated into themes. The themes were then incorporated into a concise description of the phenomenon of communication within pain management. Findings were validated by participants.

**Results:**

A total of 21 Aboriginal and Torres Strait Islander participants were involved in this study. Exploration of the communication experiences of patients and ATSIHLOs revealed overlapping themes of important barriers to and enablers of communication that affected access to care while managing pain. Acknowledging historical and cultural factors were particularly important to build trust between patients and health professionals. Some patients reported feeling stigmatized for identifying as Aboriginal and Torres Strait Islander, while others were reluctant to disclose their background for fear of not having the same opportunity for treatment. Differences in the expression of pain and the difficulty to use standard pain measurement scales were identified. Communication was described as more than the content delivered, it is visual and emotional expressed through body language, voice intonation, language and the speed of the conversation.

**Conclusion:**

Communication can significantly affect access to pain management services. Aboriginal and Torres Strait Islander patients highlighted the burden of emotional pain caused by historical factors, negative stereotypes and the fear of discrimination. Pain management services and their health professionals need to acknowledge how these factors impact patients trust and care.

## Introduction

Pain management aims to control patients’ pain or their responses to pain using multidisciplinary approaches in collaborative relationships with patients, to achieve self-efficacy and improve quality of life (1). Pain can cause physical disability, depression and lower quality of life (2, 3). Chronic or persistent pain can be an impetus for patients to seek health care (4). Because pain is a subjective and biopsychosocial phenomenon, effective pain management relies on effective communication between patients and health practitioners (5). Effective communication underpins fundamental aspects of pain care, including pain assessment, establishing management goals, and implementing treatment plans and follow-ups (6).

Among Aboriginal and Torres Strait Islander people, the burden of musculoskeletal pain conditions is 1.4 times higher than that of the non-Indigenous population. Aboriginal and Torres Strait Islander people are at a higher risk of disabling musculoskeletal pain due to a high prevalence of health, lifestyle and psychological risk factors for pain. These factors result in significant disparities in pain management outcomes (7, 8). For Aboriginal and Torres Strait Islander people, there are many barriers to access care, including geographical isolation, social-economic disadvantages, and poor experiences with health care (9, 10). The determinants of health for Aboriginal and Torres Strait Islander peoples are a result of the impact of colonisation in their communities and culture (11). The destruction of culture with significant disempowerment and marginalisation had a widespread effect on the physical and mental health of Aboriginal and Torres Strait Islander people (12). Poverty and racism have been associated with adverse health outcomes and discrimination (unjust treatment) that can occur at any stage of the lifespan (13). A recent nationwide study involving Aboriginal and Torres Strait Islanders people found that 58.5% of participants reported experiencing discrimination. Discrimination was significantly associated with diverse impacts on social and emotional wellbeing, culture and identity, health behaviour, and health outcomes. Moreover, these impacts were greatest amongst those reporting moderate-high levels of discrimination (14). This association between discrimination and health outcomes is important in the context of differences in life expectancies between Aboriginal and Torres Strait Islander people and non-Indigenous Australians highlighting the need to address discrimination as part of reducing this gap (15).

Ineffective communication is also a major barrier for Aboriginal and Torres Strait Islander people to access health care to manage their pain (5, 16). A study exploring communication experiences in pain management of Aboriginal people in two rural communities found that participants experienced discrimination from health care providers which consequently deterred people from seeking care for their pain (17). It has also been found that there is a lack of awareness and an acceptance of a deficient cross-cultural communication as the norm (18, 19). The extent of miscommunication and the potential health impact for Aboriginal and Torres Strait Islander patients is often not well understood by either health care providers or patients (20). A review of the needs of Aboriginal people with musculoskeletal pain demonstrated that focussing on improving patients' experiences of care, in particular with regards to patient-provider communication, is a crucial part of increasing access to care, by ensuring Aboriginal and Torres Strait Islander people can trust pain providers (8).

To understand the role of communication in detail, this study was designed to examine the communication experiences of Aboriginal and Torres Strait Islander patients managing persistent pain and Aboriginal and Torres Strait Islander Hospital Liaison Officers (ATSIHLO) who support these patients, aiming to identify how communication affected patients’ pain management and their communication preferences.

## Methods

### Study design

The present study is part of the first phase of a multi-centre interventional feasibility study to improve communication between health professionals and Aboriginal and Torres Strait Islander patients accessing persistent pain management services in Queensland, Australia. Details of the study protocol have been previously described (21).

The current study reports an analysis of the communication experiences of patients managing pain and of Aboriginal and Torres Strait Islander Hospital Liaison Officers supporting patients managing pain. The findings of the health professionals' perspectives of communication needs and training preferences have been previously published (22).

### Study participants and settings

A purposive sampling approach was used in the study. A service-based researcher (AC, JPK, MT and MB) at each study site identified potential participants. One participant group were Aboriginal and Torres Strait Islander patients attending three (2 metropolitan and one regional) of the five publicly-funded adult persistent pain services in Queensland. A second participant group were Aboriginal and Torres Strait Islander Hospital Liaison Officers (ATSIHLOs) working at two study sites (one metropolitan and one regional).

The persistent pain clinics are hospital-based, providing outpatient and inpatient care for people with complex pain who require a multidisciplinary approach to management. According to services audit data, Aboriginal and Torres Strait Islander patients represent between 4%–8% of the total number of patients supported by the three services involved in this study. The ATSIHLOs are Aboriginal and Torres Strait Islander people working in hospitals. Their role is to support and advocate for Aboriginal and Torres Strait Islander patients and/or their families and act as a cultural broker between the patient/their family and health care services.

### Access to the persistent pain clinics and multidisciplinary clinical interactions

The persistent pain clinics provide tailored care to patients within a biopsychosocial framework. These services aim to rehabilitate and promote self-management. Access to these services depends upon a referral usually from a general practitioner (GP). After patients are referred to the pain clinic, they are assessed according to clinical urgency following the Clinical Prioritisation Criteria (CPC): category 1 (urgent appointment within 30 days), category 2 (appointment within 90 days) and category 3 (appointment within 365 days). The first clinical interaction will involve the patient assessment by a specialist pain physician, sometimes joined by another member of the multidisciplinary team (e.g., physiotherapist, nurse, and psychologist). This interaction may take 1–2 h and options for treatment and pain management will be discussed. A patient may require adjustments to the medication, allied health assessments (e.g., physiotherapy, psychology and occupational therapy), group therapy or surgical procedures. Individual management paths vary greatly but at minimum, patients will be supported during a limited timeframe (approximately 12 months) and will be encouraged to take an active role in learning self-management strategies that address their pain.

### Data generation

Patients were initially invited to participate in a focus group. For patients who were unable to attend the focus group, in-depth interviews were offered. Patients' and ATSIHLOs data were generated through focus groups or in-depth interviews during the period of October-December 2020. An interview guide was used to ensure the interviews explored the communication experiences of patients managing pain and the experiences of ATSIHLOs working at the study sites supporting these patients. The focus groups were facilitated by an Aboriginal researchers (GP, JI), supported by an Aboriginal research assistant (KH or MT) and two non-Indigenous researchers (CMB and CJ). The in-depth interviews were conducted by two Aboriginal interviewers (MT and KH) and supported by a non-Indigenous researcher (CMB). The focus groups and in-depth interviews were audio-recorded, transcribed verbatim and imported to Microsoft Excel. Separately, the interviewers (CMB, MT and KH) used the Microsoft Excel files for analysis.

### Data analysis

This study adopted a descriptive phenomenological methodology, as described by Colaizzi (1978) (23, 24), to explore the lived experience of communication within pain management at three pain clinics. Colaizzi's method includes seven distinctive steps, six of them with an extensive description of the phenomenon under study (familiarisation, identifying significant statements, formulating meanings, clustering themes, developing an exhaustive description, and producing a fundamental structure) and a final step of validating findings from the participants who provided the data. The data generated through the focus groups and interviews with participants were reviewed several times by two researchers (CMB, KH). Each researcher independently created a table comprised of relevant statements (patient quotes). These statements were subsequently formulated into meanings and then grouped into thematic clusters. The researchers discussed their findings before identifying the main themes from the data. Each researcher then used the identified themes to develop several descriptions about the findings. These exhaustive descriptions were then revised and condensed until a final concise statement was reached. This statement was then sent to two participants from each group (i.e., patients and ATSIHLOs) for validation.

### Researchers' characteristics and reflexivity

The research team was composed of researchers from diverse academic disciplines, including medicine, nursing, psychology and physiotherapy. The team includes both, non-clinician researchers (8) and clinicians-researchers (5). The team is also comprised of researchers from diverse cultural and linguistic backgrounds. Five of the authors are of Aboriginal or Torres Strait Islander background and all the remaining authors are non-Indigenous. The research team composition promoted reflexivity across the research process, by including Aboriginal and Torres Strait Islander as well as non-Indigenous perspectives, clinical and non-clinical perspectives, and multidisciplinary perspectives.

## Results

### Participants' characteristics

Although the communication experiences reported here were all related to pain management, these experiences were not limited to persistent pain clinic services. Participants chose to describe their communication experiences across various settings such as emergency departments, general practice appointments, aged-care, or inpatient hospital care.

A total of 21 Aboriginal and Torres Strait Islander participants, 13 patients and 8 ATSIHLOs, were involved in this study. Participants were similar in their demographic characteristics.

Patients participated in one focus group (*N* = 3) and in-depth interviews (*N* = 10). Patients were more frequently Aboriginal (92%) than Torres Strait Islander, aged on average 49 years (SD = 10.34) and more than half were males (54%) (Table 1). Patients lived with pain for a median time of 10 years (range 4–55 years) and more than half (54%) were managing multi-site pain issues.

**Table 1:** Participants’ characteristics, patients time dealing with pain and Aboriginal and Torres Hospital Liaison Officers working experience.

	Patient		ATSIHLO	
Participant characteristics	N	%	N	%
	13	100	8	100
**Indigenous Status**				
Aboriginal not Torres Strait Islander	12	92	7	87
Torres Strait Islander	–	–	1	13
Both	1	8	–	–
**Age M (SD)**	49 (10.34)		51 (13.73)	
25–35 years	1	8	1	13
36–46 years	4	31	2	25
47–57 years	4	31	3	38
≥58 years	4	31	2	25
**Sex**				
Male	7	54	1	13
Female	6	46	7	87
**Time dealing with pain in years Mdn (IQR)**	10 (9.00)		–	
**Pain location**				
Back pain	4	31	–	–
Limb pain	2	15	–	–
Multi-site pain	7	54	–	–
**Working experience in years M (SD)**	–		9 (6.52)	

The ATSIHLOs participated in a focus group (*n* = 6) and in-depth interviews (*N* = 2). A high proportion of ATSIHLOs were Aboriginal (87%), on average 51 years (SD = 13.73) and were working on average 9 years (SD = 6.52) in this role.

### Aboriginal and Torres Strait Islander hospital liaison officers' experiences and perceptions of communication: identified themes

Table 2 shows the process of formulating meanings and clusters of the data derived from focus groups and interviews with ATSIHLOs. Through this process four themes and nine subthemes were identified. Theme 1 was clustered around “*the role of ATSIHLOs”* and had three subthemes: a) patients are not heard, b) ATSIHLO are the voice, support and empowerment of patients, and c) information credibility. In theme 2, “*what is communication”*, participants described their perception of communication and had two subthemes: (a) language and delivery of the message, and (b) elements of communication. Theme 3, was clustered around “*historical and cultural factors influencing communication”* and had three subthemes: (a) cultural factors; (b) historical factors (c) communication preferences; Theme 4, was clustered on “*information and literacy”* and had two subthemes: (a) sharing good stories and (b) increasing awareness about services. Supplementary Appendix S1 contains a final exhaustive description of ATSIHLOs’ experiences of communication that is based on the analysis of data generated for this study.

**Table 2:** Process of formulating meanings and clusters for the Aboriginal and Torres Strait Islander hospital liaison officers (ATSIHLOs).


Location of significant statement	Statement (quotes)	Formulated meanings	Theme cluster	Theme [T]
ATSIHLO6003 P5	“You know, in communication barriers sometimes to it”s a matter that doctors need to really listen. Sometimes you get doctors who think they know better that the patient, but no one can know what someone else”s pain is because they”re not living in their body, are they?”			
ATSIHLO6001 P2	“And most of the patients would turn around and say, you know, like I“ve told them how I”m feeling but I feel as if I don’t get heard.’	Patients are not heard/not listen to		
P3 P3	‘The health professionals, they never listen. It's like he (‘patient’) says as if they (‘health professionals’) think “I”m imagining it” or or, you know, like that sense, that he (‘patient’) feels there's no help at the hospital. So every time he (‘patient’) comes here, it's like he's expecting that from consultants that they won’t listen to him.’ ‘The patient said to me: ‘I just I just want them to listen to me. I just want them to look into it like why I’m having all this pain.’		ATSIHLOs promote patient engagement with the service. ATSIHLOs provide support, validation and comfort for patients	Role of ATSIHLOs [T1]
ATSIHLO6003 P1	‘Patient becomes unable to advocate for themselves when they’re in pain.’			
P3 P7-8	‘I think is the most frequent issue (‘pain relief’) on which I experience a patient needs advocacy, on every single care, they are not complaining for nothing.’ ‘Early years when I came here, Aboriginal people were less likely to present to hospital when they’re not well and they’d only come when seriously ill. But that has improved over the years, and we hope that it's because they know that they got us to lean on when they come in.’	ATSIHLOs are the voice and empowerment of patients (Patients in pain cannot advocate for themselves) & offers support		
ATSIHLO6003 P3	‘There was an argument there between the patient and an ED nurse over the issue of, you know, whether or not she actually had this pain.’			
P5 P7	‘…And there's another issue that comes up, too, is sometimes when the patients are asking for stronger pain relief, there's this issue that whether the drug seeking or genuine.’			
R4 P9	‘At the same time, they (‘patients’) might feel a bit of a cringe factor when they’re talking to doctors. All right. It's a case of what is this doctor going to believe? What I’m saying? Am I? Am I being a winger or those kind of factors?’ …’when it comes to pain, there's almost a blanket notion sometimes that you're only here for certain things. And, you know, from my personal experience, it was actually quite insulting to have this insistence that I was here, for particular reasons when I was actually generally unwell and in extensive pain…. I think that that again, comes down to racist preconceived ideologies that you know, sometimes we carry subconsciously.’	Credibility/stigma/validation/apprehension about the information provided: is it genuine?		
ATSIHLO6003 P6	‘But she (‘patient’) comes in always wanting (‘medication’), you know, she got very offended when I first met her about the fact her management plan referred to her opioid dependence. So I had to talk to the clinical care coordinator for the ED and said: look, you just going to have to use a different language because the impression the patient has is that you’re calling her - drug seeking.’			
P8	‘Talking to doctors, doctors need to be very careful to avoid any sense, any intuition, intuitive feeling of being judgemental about the way the person lives their lives.’	The language used and how messages are delivered by clinicians	The elements involved when communicating with Aboriginal and Torres Strait Islander patients	What is communication [T2]
ATSIHLO6001 P4	‘Okay, they (‘patients’) will say yes, yes. But afterwards, the consultant goes, the patient will turn around and say to me, I didn’t understand a bit. I don’t know what he (‘consultant’) is talking about. So then I have to explain.’			
P4 P5	‘Because the communication side of it, sometimes they (‘health professionals’) will not ask the questions directly. Others do, others don’t.’ ‘Because sometimes I (‘ATSIHLO’) go back to the consultant and I (‘ATSIHLO’) tell the consultant if I (‘ATSIHLO’) was to explain what you've just said in there to the patient. In simple terms, because they (‘consultants’) are asking questions or not understanding how to ask questions; how would you (‘consultant’) want me (‘ATSIHLO’) to explain? How would I (‘ATSIHLO’) say it? Yeah, sometimes I			
	(‘ATSIHLO’) have to go back to the consultant because it's depending of the importance. The message they’re trying to get across.’	ATSIHLO: not just verbal but visual (P15, P16, P17), tone (P6, P7, P8, P17), body language (P4, P5, P6, P7, P8, P12, P13, 17) and emotional P17		
R4 P8	‘One patient in particular, it was almost, because she, she presented so frequently. It's like, she could read everyone straightaway, you know, she wasn’t being taken seriously. And that's in spite, you know, whatever treatment she's getting through the clinic. But I feel like, again, with the tone and the body language, she's reading it straightaway, as soon as she's coming in.’			
RI2 P17	‘I think we agree that communication is about building rapport, it's about knowing the individual as they are and getting to know that person. We agree that communication is not just verbal, it's visual and it's a body language, a physical thing, and it's an emotional thing. Your tone of voice.’			
R3 P6	‘It's the tone that you know, they might be saying something nice, but how they deliver that message. Yeah, sometimes it was bossy, or really abrupt.’			
ATSIHLO6001 P4	‘You know, sometimes, sometimes just for the sake of it, or sometimes they will say, yes, you know, understood where there is a doctor telling them their medical condition. Okay, they (‘patients’) will say yes, yes. But afterwards, the consultant goes, the patient will turn around and say to me, I didn’t understand a bit. I don’t know what he (‘consultant’) is talking about.’	Cultural factor affecting communication	Cultural factors: Not asking questions Against confrontation Too impersonal Protocols Preferences	
P5	‘…Sometimes it's that shy factor, they (‘patients’) will shy away, especially when they’re not comfortable; they’re not comfortable as it is in this environment. So they’ll sort of withdraw a bit.’	Cultural and environmental factors affecting communication		
P9	‘So it's a bit some of the Indigenous families of patients that come in. When you go straight into business, they switch off straight away. If you’re talking about something that they don’t understand. There's no one there for them to explain as the doctor is going along. They’ll switch off and they will just say that yes, yes, yes, thing just so the doctor can go away.’	Cultural factor affecting communication	Historical factors: No challenge to authority Institutions are scary Consent	
**R1 P15** **R1 P3** **R4 P11** **I2 P11** **RI2 P10**	‘A lot of people, they will come through us, and they won’t tell clinicians that they are in pain, but they’ll happily tell someone like us as a third party, that they are in pain. And that's is just the way sometimes Aboriginal and Torres Strait Islander people are, is that they’re happy to go through a third party to tell that they are in pain.’ ‘I experience a lot of Aboriginal and Torres Strait Islander people, especially a lot of our elders, who are looking after young children, they have a strong threshold of pain, because it's sometimes not their priority to, to come to hospital and deal with pain, if they can, if they can deal with that pain and the threshold of it.’ ‘The fact that a lot of missions and even up to the Torres Strait Christianity is even outside of just culture alone and you have to add a layer of religion where you know that Christianity tells them like this is inappropriate, I would prefer this sort of stuff. So those sensitivities on top of culture as well.’ ‘But I guess as Aboriginal and Torres Strait Islander people, we carry different things. Every human carries different pain. We have intergenerational trauma, we have that emotional pain. And when we learn in different families, we’ve learned different ways to manage our pain, a different way to respond to pain.’ ‘I think here is like, not disregarding that as well, like culturally, for me as a younger female speaking to an older male. I personally feel intimidated, because I feel like this is not business that I should be talking about…And so I went to the gentleman, I said, I know this is an uncomfortable situation for you. And I, and I wouldn't generally come and see somebody for this because of where I'm from. And so it doesn't sit right with me. But the doctor has this concern are you all right to chat about that. And in that instant, he was like, yes, that's okay, we'll yarn about it. And he was sharing, and I was sharing too, un-shamed. And so acknowledging that, that is a cultural thing, rather than just saying, Oh, you don't have, we don’t have a male here’.	Preference of communication Cultural factor affecting communication (Pain expression and not a priority) Religion factor affecting communication Historical factors and pain expression Cultural protocols and communication preferences that exist sometimes cannot be addressed but should be acknowledged. Patient have to agree/consent		Historical & cultural factors influencing communication [T3]
**R1 P3** **R4 P14** **R4 P9**	‘I think if we want if we want our Aboriginal and Torres Strait Islander people to be a part of pain clinic, and we need to share good stories that come out of pain clinic, you know that, because it's not really something like I said, that's known to the client cohort of our people. So sharing good stories about how pain clinic has actually helped and assisted these people. And sharing those stories could actually invite more Aboriginal Torres Strait Islander people into that environment, if that makes any sense.’ ‘What's the difference about pain management and the difference in the pain clinic to dealing with managing pain with medication? And I think a lot of our mob don’t know what the service delivery is in in pain clinics. So which makes them really apprehensive to attend.’ ‘A lot of it again is fear, which is why we have a lot of fail to attend outpatient appointments or pain clinic appointments because they (‘patients’) don’t know. They don’t have that understanding of why they should go or how it can better their lifestyle.’ ‘And it's not just having the training and cultural awareness training but ensuring that it's interwoven through you know, even their training as healthcare professionals, not just a once off once a year type of thing, it needs to be continuous. And it needs to reflect how, you know, the patients that they’re (‘healthcare professionals’) currently seeing and not just be like one stock standard training either.’	Informing and sharing good stories to bring people to the service Informing – increasing awareness about services and benefits - Literacy Informing – increasing understanding the benefits of attending services Healthcare professionals need to be aware and incorporated into practice	Information to improve health literacy to improve access to the pain clinic	Information and literacy [T4]

### Condensed fundamental structure of the ATSIHLOs lived experience of communication within pain management

The ATSIHLOs identified that the way information is delivered to Aboriginal and Torres Strait Islander patients can significantly impact their engagement with treatment plans and health services. Patients may disengage from a conversation if the language is too complex, impersonal or perceived as judgemental about the way a person lives their life. Communication between patients and health professionals was described as being very visual, because patients are very attentive to health professionals’ vocal intonation and non-vocal conduct, which is perceived as communicating “emotion”. ATSIHLOs highlighted the importance of health professionals be aware that they sometimes unconsciously or unintentionally give cues that imply judgement about the way people live their lives (e.g., body language or leading questions) [T2].

ATSIHLOs reflected on the fact that many patients do not have high literacy levels and consequently have limited understanding of how health services operate and what support they can offer. ATSIHLOs emphasised the importance of promoting the positive results achieved by the pain management services with patients and families to encourage people to seek care. ATSIHLOs recommended that to improve communication between patients and health professionals it is not enough for health professionals be aware of the existence of an ATSIHLO but actually offer this support service to patients. This would help to provide a supportive environment for Aboriginal and Torres Strait Islander patients [T4].

Furthermore to provide a supportive environment ATSIHLO mentioned two very strong perceptions related to information and acknowledgement of cultural protocols: the need for acknowledgement of cultural differences and demonstration of respect to cultural protocols (e.g., related to gender of the patient and health professionals); and patient consent. For example, health professionals should acknowledge to patients that they are aware of the cultural protocol with regards to gender and age group, and in a situation of absence of a health professional of the same gender or age group, confirm with the patients, that they would still be agreeable to proceed with the assessments in this situation. This would demonstrate acknowledgement and respect to cultural protocols [T3].

Exploration of the communication experiences of ATSIHLOs revealed the important role these professionals have while supporting patients in managing pain. ATSIHLOs are pivotal in reducing tensions and increasing the trust between the patient-clinician. ATSIHLOs identified that of all Aboriginal and Torres Strait Islander patients, those living with persistent pain would be the most likely to require ATSIHLOs support. Patients in pain experience vulnerability, are unable to advocate for themselves and feel that they are not heard. There is frequently a tension from patients trying to have their pain validated but feeling that health professionals do not believe their experience of pain [T1].

### Aboriginal and Torres Strait Islander patient's experiences and perceptions of communication: identified themes

Table 3 presents the process of formulating meanings and clusters of the data derived from focus groups and interviews with patients. Six themes and 15 sub-themes were identified: (i) ATSIHLOs' role of providing assurance through their physical presence and listening to patients' needs; (ii) emotional pain: caused by stereotypes, discrimination, feeling alone and not being believed; (iii) pain measures: scales are not appropriate to measure my pain; (iv) positive experiences of communication: trust, the health professionals know me, being able to identify, explain what was going to happen; (v) negative experiences of communication: throwing pills on issues, no alternative treatment, disregard or ignored as a person, no sensitivity to patient understanding and health literacy; and (vi) factors affecting access to care: need for more Indigenous staff, access to care and respect to cultural protocols, fear of identifying as an Aboriginal or Torres Strait Islander person. Supplementary Appendix S2 contains a final exhaustive description of patients' experiences of communication that is based on the analysis of data generated for this study.

**Table 3:** Process of formulating meanings and clusters for the Aboriginal and Torres Strait Islander patients.


Location of significant statement	Statement (quotes)	Formulated meaning	Theme Clusters	Theme [T]
1063 – Page 4	“It helped me with peace of mind because I got someone. That’s what I mean someone needs to be there with them (patients) not to be there on their own.”	Physical presence, assurance	ATSIHLOs support; they are the “brokers” between patients and health professionals	Role of ATSIHLOs [T1]
R4 – Page 13	“Well they were the pawns between the system and us. The system and its rigid protocols or policies and the Liaison Officer who got them to bend or come to their senses a little bit and then hopefully through that then this person here will climb up the ladder somewhere where policy can change.”	Listen to patient needs, links the patient to healthcare system and tries to address these patient needs		
2051 – Page 15	“Those stereotypes like even over minor things if you’re stereotype people get out of the game. Don’t come near an Aboriginal person because you’ll offend them and upset them in ways you will not understand and that was my because like I had a lot of benefits and disadvantages like I was too white for some relatives and I’m too dark for white people. So I experienced both spectrums and I know well just for myself.”	Stereotypes: too white to be black and too dark to be white	Psychological factors causing pain: stereotyping, discrimination and stigma	Emotional pain [T2]
R5- Page 18	‘Like I know that like some Indigenous patients are very or can be very self-righteous, absorbed and I really, really love the patients that go “Oh well you’re discriminating against me because I’m Aboriginal” and I turn around and go: “*Well you’ve spoken to the wrong person”*. And then they look at me and I go: “*Well you’re looking at the XX*…. *Aboriginal and Torres Strait Islander of the year*”.’	Discrimination: too white to be black		
R6- Page 18	‘You know what I say to them? *“Honey you barked up the wrong tree because I’m the only white blackfella that you’re going to meet today”*. Because my own people say “Oh you’re too white. You’re too white”. That took so much out of me growing up. Even when I got put in homes and everything else like that. …	Discrimination: too white to be black		
R3 – Page 16	It is for your own people to shun you.’	Discrimination: too white to be black		
R3 – Page 19	‘Oh you know, “These white fellas that think they’re black”.’	Discrimination for being Aboriginal		
	‘When the aged-care guy rang up to say that he was coming out to see what help I needed and then we’d been chatting away about you know, fine, and then he said, “Hang on, you’re not old enough”. And I said, “Yes I am, I’m Aboriginal”, straight away he goes “Right, how many people live in the house? Anyone fresh out of jail? Is there any guns? Is there any vicious …”.’			
R5 – Page 21	‘Straight in to the judgement. You know straight in to the whole well they look at you, they look at me and go “Oh well you must be a smoker and drinker”.’	Discrimination for being Aboriginal		
R6 – page 22	“So the stigma that surrounds Aboriginal people, Aboriginal people that have had something go wrong in their past or anything is absolutely atrocious. And these people should be taught while they’re in university that you have to look beyond that, you have to look at the person, you have to look at that person now not their past. Yes, have a look at their medical past, get to know that person, ask your questions, do it in a caring one-on-one level of emotional connection rather than a clinical piece of paper and a pen.”	Discrimination for being Aboriginal		
R5 – Page 7	‘“Oh well you’re just drug-seeking”.’	Stigma – drug seeker		
R3 – Page 8	‘“You’re a doctor shopper.”’	Stigma – drug seeker		
2064 – Pages 3–4	‘I got the vibe that she thought that I was abusing drugs the way she approached me. So I walked out of the clinic, they all came chasing after me and they said “What’s wrong? What’s wrong”? And I said “This is where the communication breaks down and you are jumping to conclusions without actually approaching me in a proper mannered way”. So and I explained to that person I said “Look there are drug abusers with medication but you had a chance to actually ring my doctor’s office or whatever the medical centre that I’d been going to. Not trying to”….yeah she was trying to gouge out something that she thought that I was trying to just abuse the drugs. Which actually made me really, it makes you really bad and sad and emotional and all those things. And then with me I had, I made sure I put in a full report so those things don't happen again to other patients. But you’ll get, often those things will happen, especially when you’re on pain drugs.’	Stigma – drug seeker		
R5 – Page 9	‘I think it’s the fact that usually you get to, patients get to desperation stages with pain and patients just want to be believed.’	Want to be believed/validated		
1108 – Page 1	‘People like to call you a drug fisher or a hypochondriac that your pain couldn't possibly be that bad. And you’re still able to do things with your children day to day things. Well they need to be done and yes I am in that much pain.’	Stigma – drug seeker and not believed		
2067 – Page 1	‘They just that was just the way the conversation was headed. And I mean they weren’t nasty about it or anything but I just came away from it feeling left out in the cold I guess.’	Feeling isolated and alone, discharged per protocol not because patient was well		
R6 – Page 22	‘And I really hate when they say “On a scale of one to ten what’s your pain like?” I said, “Honey for you city people mine’s a nineteen”. I said “I was born country, I’ll handle it til it hits thirty, thanks”. The scale of one to ten is just bulls*** because you cannot put a scale on pain because some people mask that pain better than others.’	Pain measures do not appear to assess appropriately pain for Aboriginal and Torres Strait Islander patients	Scales do not measure patient pain	Failure in pain measure [T3]
1104 – Page 2	‘I sometimes feel that they don't understand where I'm coming from with the pain. I feel like, sometimes I feel like they’ve just got me in that category where everybody suffers the same pain from an injury.’	My pain is not in a category	No recognition of individual experience of pain	
2064 – Page 4	‘They were trying to get information out of you but when you were in pain and you come up with the wrong answer then she was punishing me for that and then saying “Well what’s the story? You’re not getting your story straight”.’	High levels of pain interfere in the ability to think clearly and describe or measure pain	Ability to thinking is compromised affecting response	
2067 – Page 2	‘…places and appointments and try and keep them all straight in my head. And on a calendar and all the rest of it and I'm not good with those sorts of things anyway. But when I'm feeling you know when my pain levels are particularly high and it interferes with my ability to think clearly.’			
1054 – Page 1	‘Well I …I have found myself quite a good doctor at the moment. He is Sri Lankan by birth, he’s got a Russian wife, he’s very culturally aware of different cultures. And he respects my cultures and my beliefs.	HP respects patients’ culture and beliefs	Enablers of effective communication: Respect	Positive communication experiences [T4]
	He doesn’t push …he doesn’t push the hard line drugs on me.			
	He’s respectful and he will say to try herbs and vitamins before all different sort of things before he pushes the hard line drugs on me and to me.’	HP offers alternative treatments	Trust	
R6 – Page 14	‘Then don’t be clinical. There’s no need to be clinical, if you’ve got fifteen minutes with this person and you’re going through these questions and everything else like that pull up. Okay here’s the first question, “Oh you know Aunty how are you feeling? I’ve got a couple of questions here, we might just go through them a little bit slow about what’s happening” and you know make conversation as well as get the questions answered. And that person’s going to think, “Shit they’re taking time”. It doesn’t have to be an hour, it’s how you put the question across, it’s how you communicate with that person while you’re doing what you need to do.’	Take the time, is not clinical, get to know the patient	Showing interest Offering support Working together	
1054 – Page 2	‘Well mainly my current psychologist has asked if I would like to see an Aboriginal counsellor. Instead of me being comfortable with just an ordinary so called Caucasian counsellor. They’ve actually suggested if I wanted to that I could see an Indigenous counsellor, Indigenous psychologist. Which that actually blew me away when they offered me that. I said I didn't mind either way, anybody.	Health professional offers Indigenous support services		
	Yeah. And they asked if I would be more comfortable with an Indigenous sort of psychologist or counsellor. Which I think that totally blew me away.’			
2064 – page 5	‘Sometimes in the big hospital it’s always good to ask would you like me to call an Aboriginal liaison officer to sit in with or like to give them information that says “Look if you feel uncomfortable or felt that this didn't go too well you can contact the Aboriginal liaison officer”. And I think that’s the other thing that they should be doing is getting the patients the option of that.’	Health professional offers Indigenous support services		
1063 – Page 2	‘I have a doctor and I explained to him what was going on and there’s not many doctors who would do that. And I said to him I said no doctor is listening to me but I need to know that I can bend and stretch and actually do things with my grandchildren I couldn’t even do any of that until he listened to me.’	Listen to me		
1108 – Page 3	‘Yeah…my doctor was good. She listens to everything that I'm saying and it’s because she listens that I’ve avoided a lumbar puncture in so long. And did she do anything in particular that you know she’s listening to you? No, it’s basic you know body language, eye contact	Listen to me, body language, eye contact		
1062 – Page 2	I don't think it’s just me being Aboriginal I think it would be a lot of Aboriginals would have the same anxiety with people not listening to them.’			
2071 – Page 9	‘He (doctor) was absolutely brilliant he’d sit down and discuss everything. And then when he found out what it was he’d sit down and discuss it with me and my partner, like my wife and you know discuss this is what’s going to happen. You know it could come, it could like it’s there it could go away you know it may stick with you for the rest of your life.’	Sit down, explained and discussed what is going to happen		
1104 – Page 4	‘But the one-on-one with Doctor XX, it was brilliant, it was amazing. Because she just sat there and listened and we talked. Yeah you know she pulled up a chair, she wasn’t there on her computer going, “All right Mister XX has had blah, blah, blah” that was it. You know she pulled up a chair, sat down, we had a yarn. The other doctors like the young doctors stayed with her and we just yarned about everything. She wanted to know more about our culture so I taught her. And so she said that opened a door up for her.’	Sit down and had a yarn, showed interest in knowing more about patients’ culture		
1063 – Page 2	‘Yeah when I see the regular doctor (XX) because I was just saying she’s very good, very empathetic, understanding, she understands probably the best out of the lot. She’s been really good.’	Empathetic, understanding		
2064 – Page 2	‘Well when I go see my doctor it takes me time to see if I really trust him or not. But when he’d sat down and spoken to me and listened to me that’s when I knew I could trust him with any problem with my back.’	Building trust		
1063 – Page 3	‘With me is the, I always return to resources. So if you’ve got the right resources like you’ve got them on a piece of paper sent to you via email then if you’re having problems you can always go back and have a look at it again. Or you can show a family member or whoever, you might be able to show your GP what you’re doing. So that communication link, that’s one of the best things.’	Resources can help the patient re-visit information. Helps to explain to the family		
	‘See it works both ways when it comes to the patient they’ve got to learn that it’s not going to be finished overnight it takes time. Especially the doctor you’ve got to let him know maybe a month’s time I don’t know I cannot promise you.’	Collaboration & understanding		
1054 – Page 3	‘Oh well one doctor that I was seeing was throwing pills at my issues. And one time during a consultation he took a private mobile call, a private call on his mobile and just had a talk to the person on the other end for five minutes with me sitting in the chair twiddling my thumbs so to speak. He was just totally disrespectful and I haven’t seen him since.’	Disregarding the patient	Barriers to effective communication: Repeating stories Multiple doctors No communication between team members No consideration of the understanding of patients	Negative communication experience [T5]
1062 – Page 3	‘Well okay a couple of the doctors I’ve just walked in there and I said you know “I need my scripts filled”. And that’s it they just fill them and you know when you’re getting a hundred and twenty milligrams of OxyContin you know they’re not even asking questions. Like they say “What’s it for”? Oh “Pain” and that’s it they’re just quite happy just to write it out, write out any prescription drug for you.	Are not interested in your health, just to give prescriptions		
1104 – Page 4	They’re the only two doctors that I’ve had that have showed any interest in my health at all.’	Regular doctors vs. temporary, they don’t know you, and don’t provide the same care		
	‘Yeah I’d say yes you can know the difference. Someone that I see regularly there compared to like a temp, there’s a difference. But I guess in all fairness they don't know me, they’re just there filling in, they wouldn't know my history as well. So I guess that’s probably why.’			
R5 – Page 4	‘And you know a lot of Indigenous people and Aboriginal people the way they get spoken to and it’s “Blah, blah, blah” (*talking very quickly*) and you only hear, you know especially elderly and I’m looking at my mum. She only, you know unless you have her attention you only get, she only gets parts of the information.’	No sensitivity to identify patient understanding, sending out the message, no concern about the understanding of the patient		
R5 – Page 4	‘It actually looks at the, like looking at the patient, all of the patient’s needs and we find that patients, the teams don’t talk. And the teams don’t read the other teams notes half the time and it … you know like it’s, you know when you have them saying opposite things like the patient gets confused.’	Healthcare team does not talk to each other		
R3 – Page 6	‘Well I had an experience with a rheumatologist when I felt like I wasn’t in the room. I felt like you know I was totally ignored as a person, as a patient and she didn’t … Yeah I was really shocked.’	Ignored as a person		
R5 – Page 7	‘And it gets really frustrating because literally you come down to the pain clinic here, your first one and it’s a two-hour face-to-face and they go through everything, they’ve documented everything, you know they’ve got all of your notes and everything like that. And then you see the same doctor and then you’ve got an intern or someone like that is the next one to see you and they go… then you have to go back over.’	Frustration- telling their medical history all over again		
2064 – Page 1	‘Because at the end of the day I think everyone wants to know what the hell is going on with your body even if they can try and find other words to use than those big medical ones. Try and cut it down and make it simple but understandable.’	Health literacy, medical jargons and understanding		
2062 – Page 6	‘To me to go to the root of the things, if we can get more Indigenous nurses and doctors and you know and like the hard things. In our way, we speak to one another all being more sensitive culturally to how you know like that’s why a lot of us probably die and stuff because we’re too ashamed to go to the doctors.’	Need for more Indigenous staff to access care to overcome shame	Lack of understanding of historical factors, cultural responsibilities, customs and protocols	Factors affecting access to care [T6]
2062 – Page 6	‘Yeah and see when I go to the Indigenous I tell all my doctors about they’ve got a female there for female health because you know what I mean? …	Access to care and cultural protocols, men’s and women’s business		
	And if you go, “I’m sorry but you’ve got to go and see a man” well a lot of them won’t go.’			
2071 – Page 4	‘The hospital itself doesn’t understand our culture. Whereas you know like I get put in a room you know with three women. That can’t happen. And I say to them, “I can’t be in here hey, it’s not fair. I don’t want to be in here”. Or they put you in with two white fellas and a little Murri girl in the corner and again I don’t want to be in this room. You know they’ve got to understand our culture.’			
2062 – Page 6	‘Yeah so maybe when they’re coming through the universities and they’re at TAFE and that maybe they need to be taught how to be sensitive to Indigenous culture. I mean I don’t know what happens when you train to be a doctor and how to treat different generations you know? Like a seventy year old Aboriginal woman with someone who’s my daughter’s in her thirties you know who didn’t grow up on a mission or you know wasn’t treated … You know what I mean?’	Historical factors affecting access to care		
2051 – Page 10	‘And being Indigenous your guard’s up already. And then if you’ve been an Indigenous person brought up through institutionalisation then you’re a different fort again.’			
2064 – Page 5–6	‘Now after all the conferences that I’ve been to it is probably a mixture of people that say why do we have to tick that box? So there’s sort of like an issue with that as well why they…and then some people feel if I tick that Aboriginality box then they’re just going to not give me the best care because I'm Aboriginal.’	Fear; not identifying out of fear the patient would not have the best care		
2073 – Page 2	‘Look I mean can I tell you, when I first, about my transplant, I was too scared to say I was a blackfella and the reason was, was because I thought I would never get a transplant and that’s how I felt. Because I just felt you know they’re not going to worry about us.			
	And I’m not the only one, I mean a lot of our mob’s gone through it and a lot of your ancestors would have too. So you know it’s not easy and then so I thought, “If I don’t tell them I’m a blackfella I might have a transplant and then get a chance”.			
	Yeah I honestly thought they’d treat me differently. But I have to admit they didn’t. The unit as far as I was concerned didn’t care if I was black, green, purple or white.			

### Condensed fundamental structure of the patients lived experience of communication within pain management

Patients managing pain described their experience as of vulnerability, recounting their stories and searching for the cause of their persistent pain. In this search, patients sought for care in several services and communicated with many health professionals. This journey challenged patients to understand how they were approached by health professionals, assessed and supported. Patients confirmed the “broker” role of ATSIHLOs in breaking down communication barriers and linking patients to the service. The simple fact of having the presence of an ATSIHLO gave some patients the assurance of a safer environment [T1].

Patients described that their physical pain would sometimes be secondary to family and community commitments and responsibilities. Being in pain affected patients' response to assessments, and sometimes they felt their responses did not align with the expectation of the health professional. This would cause tension, mistrust and a feeling that patients were not heard by health professionals. Patients described feeling emotional pain for being discriminated and stigmatised. This included discrimination on the basis of being an Aboriginal or Torres Strait Islander person. Conversations would change just by the fact that the patient disclosed their background and questions would be directed to issues with drugs and alcohol misuse, and incarceration. For some, it also included discrimination on the basis of not having a stereotypical physical appearance of an Aboriginal or Torres Strait Islander person (e.g., fair skin). Lack of acceptance of patients' Indigeneity, fear of less opportunities for care and a stigma of being seen as a “drug seeker”, combined with the shame of sometimes not disclosing their Aboriginal or Torres Strait Islander background, caused significant emotional pain to patients. Patients' trust that health professionals would help in the management of their pain was increased when patients felt heard. In these situations, patients felt respected and would disclose more details about themselves [T2].

Positive communication experiences were reported when the health professional invested more time exploring patients' cultural background and beliefs, recommended alternative treatments (e.g., vitamins, traditional or herbal treatments) and offered Aboriginal and Torres Strait Islander support services (ATSIHLOs or community services) [T4].

In contrast to the positive experiences that were reported, patients perceived that their pain management would be compromised when health professionals would not communicate with them, and would continuously prescribe medication without verifying how the patients were feeling. These health professionals were perceived as “throwing pills on issues” and disregarding the patients' health. This affected patients' ability to physically function, to work and to be active [T5].

Factors such as increasing the number of Aboriginal and Torres Strait Islander staff, patient age, health literacy and cultural protocols (e.g., men's and women's business) should be observed during consultations to improve communication. This was especially relevant to elderly patients living in a more traditional cultural environment, who felt extremely challenged to manage pain and navigate an environment that does not recognize their protocols and customs with regards to age, gender and role in society as an Elder [T6].

## Discussion

This study explored patients' and ATSIHLOs' communication experiences across different settings of the healthcare system while managing pain or supporting patients with pain. Although previous studies have explored communication and barriers to management of pain among Aboriginal and Torres Strait Islander patients (5, 17), this study describes the phenomenon of patients' preferences for communication and ways communication affected their pain management. The main findings of the study is that effective communication for Aboriginal and Torres Strait Islander patients in the pain management depends fundamentally on trust, based on the understanding of the impact of emotional pain caused by historical factors, stereotyping and discrimination, and by acknowledging cultural protocols. It is well understood that Aboriginal and Torres Strait Islander understandings of health are typically broader than those held by non-Indigenous Australians and this holistic understanding is not well accommodated by mainstream healthcare services. The current study identifies important factors for holistically treating Aboriginal and Torres Strait Islander patients (25). Some of these findings are specific to Aboriginal and Torres Strait Islander patients experiencing pain, while others may be transferrable to Aboriginal and Torres Strait Islander patients more generally.

Trust is built when patients feel that the health professionals know them or that they want to know about them. When health professionals actively listen and learn about the patient, that patient is more likely to trust them. In this context, actively listening often requires understanding the factors underpinning the extent to which Aboriginal and Torres Strait Islanders engage with health services (e.g., historical factors, racism/discrimination, and protocols around gender).

Patient perceptions of not being heard or believed and consequently feeling that they have ongoing untreated pain with results that were below their expectations are findings shared by other studies amongst non-Indigenous participants living with persistent pain (26, 27). However, for Aboriginal and Torres Strait Islander patients this situation appears to be aggravated by the perception of persistent disregard of them as individuals. This perception links back to the historical events of colonisation and struggle for recognition of Aboriginal and Torres Strait Islander identity (28). Further, some of the frustration of not being heard reported by patients may be caused by the complex and subjective nature of pain (29). Health professionals argue that many persistent pain conditions are of non-specific origin, are difficult to differentiate given the diagnostic criteria and diagnosis based heavily on exclusion (30). Additionally, pain may be dismissed or overlooked as someone else's responsibility. Health professionals, especially in primary care, may have time constraints during standard consultations and pain management may be an “add-on” to assessing and managing other chronic diseases. Specialists outside pain medicine may feel it is not their area of expertise. The multidimensional nature of persistent pain and challenges in managing it in generalist health care settings are barriers to optimal pain care (26).

Particular to Aboriginal and Torres Strait Islander patients managing pain is the frustration and emotional pain caused by historical discrimination, stereotypes and stigma. Patients reported enduring emotional pain during some interactions with health professionals and support services. Some of the emotional pain resulted from stereotypes about what the physical appearance of an Aboriginal and Torres Strait Islander person should be. Patients with fair skin reported being questioned and not being accepted as being Aboriginal or Torres Strait Islander. According to Paradies (13) racism can be expressed through stereotypes (cognition), prejudice (emotions) or discrimination (behaviours). The questioning of patients Indigeneity due to their skin colour made patients feel that did not belong and for some, resulted in considerable psychological distress. Research on Aboriginal mental health and wellbeing highlights the importance of a strong connection to culture and pride about Aboriginal identity (31). A positive cultural identity can impact on the individual sense of belonging, social support and self-confidence (32). Another cause of emotional pain for Aboriginal and Torres Strait Islander patients was the fear of discrimination. Patients described an intense internal conflict between deciding to disclose their Indigeneity, and face the risk of not receiving the same treatment as other patients, or feeling guilty for not acknowledging their background. Some patients reported that there was an instant change in the conversation prompted by their background disclosure which caused them significant psychological distress.

Discrimination or stereotype threat has been found to impair performance by inducing psychological distress, poorer mental health and decreased life satisfaction (33). Persistent exposure to stereotype threat may result in avoidance or withdrawal from the threatening situation. The process begins with patient awareness that they belong to a group negatively stereotyped, making them more vigilant for cues that confirm the negative stereotype. For Aboriginal and Torres Strait Islander people discrimination was found to be associated with negative outcomes in physical and emotional well-being, and was similar when discrimination was attributed to Indigeneity (i.e., racial discrimination) or when discrimination was not attributed to Indigeneity. However, attribution to Indigeneity was more frequently reported by people experiencing moderate-high compared to low levels of discrimination (90.6% vs. 63.5%, respectively) (14). While the costs of racism and discrimination have been well documented, it remains less clear in the Australian context what predicts attitudes and behaviours that affect negatively the outcomes for Aboriginal and Torres Strait Islanders people (34, 35). Implicit prejudice (“unintentional”) appears to cause more damage than deliberate prejudice given that discrimination is experienced in a variety of contexts (e.g., labour market, criminal justice, and housing).^34^With regards to pain, there was a variation of attribution when examining the association between discrimination and outcome (14). Attribution is difficult, as individuals may have multiple characteristics for which they may be disadvantaged (e.g., race, gender, age, and socio-economic status) and the interaction of these characteristics influence the type of experiences an individual has and inequalities (36). Further research is required to better understand why a divergent pattern emerged for pain.

Discrimination and stereotypes can also affect communication. When the communication between patients and health professionals is not perceived as respectful and pleasant, patient health outcomes will most likely be impacted (37). An Australian longitudinal study exploring the health system navigation by marginalized groups (i.e., people living in rural or remote areas, sexuality and/or gender diverse, refugee, homeless, and/or Aboriginal) identified that participants perceived and experienced multiple forms of discrimination impacting on their access to care and contributing for further marginalization. Many participants reported that health professionals failed to understand how multiple disadvantage could affect their ability to navigate through the health system. The participants also indicated that access to information to improve health literacy, reduce stigma about seeking support and help in the decision making process could mitigate the impact of discrimination and stereotypes (38). Good communication between patient-provider can promote adherence to lifestyle changes, appropriate medical treatment and improve the reported experience of care (39, 40).

In this study, some patients acknowledged that their fears were unfounded and that they felt empowered by disclosing their background and were fully supported by health professionals.

With regards to pain measure, patients in this study reported being locked into a category that would not reflect their individual experience of pain. For Aboriginal and Torres Strait Islander patients historical factors related to colonization, institutionalization and separation contributes to a significant burden of emotional pain making the physical pain sometimes less important (17) and more difficult to measure with standard measurement tools (41). A literature review investigating the pain expression among Aboriginal and Torres Strait Islander peoples found that health professionals' expectations of pain expression based on predominantly Caucasian experiences may affect the interpretation of observations (16). For example, some patients would present verbal and non-verbal silence in response to pain (42). Family and community responsibilities will mostly prevail above patients own health concerns and knowledge about the pain experience and the design of culturally-relevant scales for Aboriginal and Torres Strait Islander people essential (41).

Among the enablers of communication and improvement in pain management, patients and ATSIHLOs agreed that demonstrating understanding and acknowledging cultural differences and protocols were imperative to establish trust and a collaborative relationship between health professionals and patients. An empathetic and culturally sensitive approach could reduce tension and instigate a conversation about what is relevant to the patient and not be dictated exclusively by a clinical agenda. Most of the participants agreed that when they felt listened to, they could trust the health professional. Respect for some cultural protocols regarding age and gender, e.g., men's and women's business, were mentioned by many patients. Recognising the importance of integrating cultural knowledge and contextualising this to the clinical setting, the current study informs a communication intervention designed to enhance capacity in culturally competent care for Aboriginal and Torres Strait Islander patients with pain (21).

## Conclusion

Communication can significantly affect access to pain management services. Although some of the communication experiences in pain management are common to most patients, there are specific issues for Aboriginal and Torres Strait Islander patients. Aboriginal and Torres Strait Islander patients highlighted the burden of emotional pain caused by historical factors, negative stereotypes and the fear of discrimination. Health professionals who provide pain management services need to acknowledge how these factors impact patients and their trust. A model of care that combines greater cultural understanding and fosters trust and respect between patient and health professional could mitigate emotional pain and enable pain management services that are more patient–centred and improve access and effectiveness of service.

## Data Availability

The raw data supporting the conclusions of this article will be made available by the authors, without undue reservation.
